# Survey of physician knowledge of congenital cytomegalovirus infection and clinical practices in Japan: A web-based survey

**DOI:** 10.1097/MD.0000000000027589

**Published:** 2021-11-05

**Authors:** Aya Okahashi, Masayuki Kobayashi, Kotoba Okuyama, Naomi Hiraishi, Ichiro Morioka

**Affiliations:** a Department of Pediatrics and Child Health, Nihon University School of Medicine, 30-1 Oyaguchikamicho, Itabashi-ku, Tokyo, Japan; b Medical Affairs, MSD K.K., Kitanomaru Square, 1-13-12 Kudan-kita, Chiyoda-ku, Tokyo, Japan.

**Keywords:** awareness, congenital cytomegalovirus infection, congenital infection, knowledge, physician, pregnancy

## Abstract

Congenital cytomegalovirus infection (cCMVi) can cause serious and long-term effects in newborns. Without available vaccines or antiviral prophylaxis, prevention strategies for cCMVi and cytomegalovirus disease during pregnancy are limited to hygiene and behavioral interventions to prevent transmission. The objective of this study was to assess cCMVi-related awareness, knowledge, and physicians’ actual and preferred clinical practices in Japan. This web-based cross-sectional survey was conducted using online panels. Survey invitations were sent by email to physicians (pediatricians, obstetricians, otolaryngologists, and internists). Participants were asked about their awareness of congenital conditions, including cCMVi. Participants who were aware of cCMVi were then asked additional questions related to the study objectives. Participants included 292 pediatricians, 245 obstetricians, 245 otolaryngologists, and 279 internists. Awareness of cCMVi was generally high (69.2%-97.6%). Pediatricians and obstetricians were most knowledgeable about cCMVi; however, responses to specific questions such as those pertaining to risk factors, patient counseling, and clinical management of cCMVi varied. For example, correct identification of potential cytomegalovirus transmission routes among pediatricians ranged from 36.8% to 65.6%. Survey results showed a discrepancy between responses when physicians were asked about their actual and preferred clinical practices to manage cCMVi. For example, although around 90% of obstetricians and pediatricians considered it preferred practice to educate pregnant women about cCMVi, only 60.1% of obstetricians reported being able to actually do so in current practice.

This survey revealed that knowledge about cCMVi among Japanese physicians could be improved and identified variability in clinical practice.

## 1 Introduction

Human cytomegalovirus (CMV; human herpes virus 5) is a *β*-herpesvirus that can cause serious disease in immunocompromised individuals or when acquired congenitally by mother-to-fetus transmission during pregnancy (congenital CMV infection; cCMVi).^[1]^ While most cCMVis are asymptomatic, approximately 10% to 15% of infants with cCMVi have symptoms at birth.^[2–4]^ CMV-related sequelae, which can include hearing loss, vision impairment, intellectual disability, developmental delay, microcephaly, epilepsy, and cerebral palsy,^[5–7]^ occur in 40% to 58% of infants with symptomatic infection and approximately 14% of infants with asymptomatic infection.^[3,8]^ Sensorineural hearing loss is the most common cCMVi-related sequelae^[9]^; cCMVi is responsible for 25% of sensorineural hearing loss in children at 4 years of age.^[10]^ cCMVi has an overall birth prevalence of 0.64%,^[2]^ making it one of the most common congenital infections. There are an estimated 60,000 newborns with cCMVi each year in the US and Europe combined.^[11]^ In Japan, approximately 0.26% to 0.50% of newborns have cCMVi,^[12–15]^ and the rate of late-onset sequelae is 7% to 12%, which is similar to the rates in the US and Europe.^[16]^ Hearing loss and language disability are common sequelae of cCMVi in Japan.^[16]^

Currently, there is neither an effective vaccine against CMV nor an established standard antiviral treatment, so prevention of maternal CMV infection during pregnancy by practicing hygienic measures, such as handwashing, is the best available mitigation strategy.^[17,18]^ In many countries, including Japan, awareness of cCMVi and related preventative measures is low, both among pregnant women and the general population.^[19–24]^ Studies conducted outside of Japan suggest that knowledge of cCMVi is low among physicians, including obstetricians and gynecologists.^[25–30]^ This low awareness and lack of knowledge of cCMVi presents an obstacle to the implementation of preventative measures. The window for cCMVi diagnosis is short (within the first 3 weeks of life) and treatment should be initiated within the first month of life in symptomatic patients.^[31]^

Comprehensive understanding of cCMVi among physicians, particularly those caring for pregnant women and infants, may facilitate both the prevention of maternal infection and early intervention for affected infants. The patient group of TOACH-no-Kai and a research group funded by the Agency for Medical Research and Development are working toward raising awareness on mother-to-child infection, including development and distribution of leaflets on this subject. However, to date, awareness and knowledge of cCMVi among physicians in Japan has not been well documented. Thus, the purpose of this web-based survey, which was conducted in Japan, was to assess cCMVi-related awareness, knowledge, and actual and preferred clinical practices among pediatricians, obstetricians, otolaryngologists, and internists. This information is expected to help experts and researchers identify areas for potential improvement and to help guide the development of effective strategies for addressing the burden of cCMVi in Japan.

## 2 Participants and methods

### 2.1 Participants

Potential participants were identified from an online panel of medical doctors in Japan managed by PLAMED, Inc. (Tokyo, Japan), and were randomly selected and recruited for participation. Physicians who mainly practiced in the areas of pediatrics, obstetrics, otolaryngology, and internal medicine were included. Those with <2 years of post-medical school experience were excluded.

### 2.2 Study design

This was a web-based cross-sectional survey to assess awareness, knowledge, clinical practices, experience, and attitudes and opinions about cCMVi among physicians (pediatricians, obstetricians, otolaryngologists, and internists) in Japan and was conducted using online panels. Pediatricians and obstetricians were included because they are directly involved in the education of pregnant women on mother-to-child infections, screening for such infections, and the care of infants and young children. Otolaryngologists were included because they play a leading role in the management of hearing loss, a major symptom of cCMVi. Internists were considered to be the most generalizable comparable reference as non-specialists in this area. The survey questionnaire was distributed from July 2020 to September 2020.

Potential participants received an e-mail invitation to participate, which included a link to a webpage providing study information and the option to provide electronic informed consent for participation. Consenting participants answered screening questions to confirm eligibility; those who met the inclusion criteria and none of the exclusion criteria were able to proceed to the main survey questionnaire. Eligible participants were asked to answer questions about their demographic information and awareness of congenital conditions, including cCMVi. Participants who were not aware of cCMVi ended the survey whereas those who were aware were asked additional questions regarding knowledge, actual and preferred clinical practices, attitudes and opinions, and experience related to cCMVi. We developed a new survey questionnaire based on, and using wording consistent with that in prior publications that primarily assessed cCMVi awareness, knowledge, and practices,^[21–28,30]^ taking into account the authors’ expertise and clinical experience. To minimize non-response bias, the number of questionnaire items was restricted, and participants who were unaware of cCMVi ended the survey early.

The study protocol was approved by the ethics review board of the Medical Corporation Toukeikai Kitamachi Clinic, and all participants provided electronic informed consent. The study was conducted in accordance with the Good Pharmacoepidemiology Practice^[32]^ and applicable laws and regulations.^[33]^ The study was registered at UMIN Clinical Trials Registry (UMIN000041260).

### 2.3 Survey outcomes

This survey included several outcomes: awareness of cCMVi and other congenital conditions; knowledge on potential cCMV transmission routes, signs and symptoms, long-term effects, and preventative behaviors; actual and preferred clinical practices; cCMVi-related beliefs; and awareness of educational leaflets. All participants were asked about awareness and knowledge, including awareness of educational leaflets on mother-to-child infection. Internists were asked no further questions, whereas pediatricians and obstetricians were asked about their actual and preferred practices on providing education about cCMVi to pregnant women, cCMVi screening, and how easy they thought it would be for pregnant women to perform preventative behaviors. Otolaryngologists were surveyed about newborn hearing loss screening. In addition, pediatricians were queried about follow-up for symptomatic and asymptomatic infants, and obstetricians were asked about their experience with abortion due to cCMVi.

### 2.4 Statistical methods

No power calculation to determine the sample size was performed for this descriptive survey. The target number of respondents was 200 each for obstetricians, otolaryngologists, and internists; and 250 for pediatricians.

Summary statistics (e.g., number of participants, mean, and stand deviation) were calculated for continuous variables. The number and proportion of participants within each corresponding category were calculated for categorical variables. For comparisons related to awareness of cCMVi and other congenital conditions and knowledge of cCMVi among the physician groups, analysis of variance, Pearson Chi-square, and Fisher exact tests were used. A *P* value of ≤.05 was considered statistically significant. Analyses were performed using SAS Release 9.4 (SAS Institute, Inc., Cary, NC).

## 3 Results

### 3.1 Participants

The flow chart diagram for the study is shown in Supplemental Digital Content 1. Participant characteristics are presented in Table 1. Participants included 292 pediatricians, 245 obstetricians, 245 otolaryngologists, and 279 internists; of those, 285 (97.6%), 238 (97.1%), 219 (89.4%), and 193 (69.2%), respectively, were aware of cCMVi (Table 2).

**Table 1:** Characteristics of questionnaire respondents.

	Pediatricians n = 292	Obstetricians n = 245	Otolaryngologists n = 245	Internists n = 279
	n (%)			
Sex				
Male	252 (86.3)	194 (79.2)	219 (89.4)	252 (90.3)
Female	40 (13.7)	51 (20.8)	26 (10.6)	27 (9.7)
Experience (yrs)				
2–<5	12 (4.1)	6 (2.4)	7 (2.9)	15 (5.4)
5–<10	25 (8.6)	19 (7.8)	31 (12.7)	27 (9.7)
10–<20	83 (28.4)	61 (24.9)	70 (28.6)	102 (36.6)
20–<30	76 (26.0)	66 (26.9)	73 (29.8)	82 (29.4)
≥30	96 (32.9)	93 (38.0)	64 (26.1)	53 (19.0)
Board certification				
Certified^∗^	265 (90.8)	234 (95.5)	211 (86.1)	216 (77.4)
Certified in perinatology^†^	22 (7.5)	41 (16.7)	–	–
Clinical practice setting				
Clinic (<20 beds)	82 (28.1)	62 (25.3)	94 (38.4)	70 (25.1)
Hospital (≥20 beds)	208 (71.2)	181 (73.9)	150 (61.2)	208 (74.6)
Other	2 (0.7)	2 (0.8)	1 (0.4)	1 (0.4)

**Table 2:** Awareness of congenital cytomegalovirus infection and other congenital conditions.

	Pediatricians n = 292	Obstetricians n = 245	Otolaryngologists n = 245	Internists n = 279	*P* value^∗^
	n (%)				
Congenital CMV infection	285 (97.6)	238 (97.1)	219 (89.4)	193 (69.2)	<.001
Parvovirus B19 infection	285 (97.6)	224 (91.4)	112 (45.7)	194 (69.5)	<.001
Congenital toxoplasmosis	285 (97.6)	238 (97.1)	181 (73.9)	204 (73.1)	<.001
Congenital rubella syndrome	286 (97.9)	243 (99.2)	232 (94.7)	253 (90.7)	<.001
Group B streptococcal infection	282 (96.6)	237 (96.7)	157 (64.1)	203 (72.8)	<.001
Spina bifida	286 (97.9)	242 (98.8)	172 (70.2)	221 (79.2)	<.001
Fetal alcohol syndrome	232 (79.5)	179 (73.1)	68 (27.8)	84 (30.1)	<.001
SIDS	288 (98.6)	236 (96.3)	206 (84.1)	245 (87.8)	<.001
Trisomy 21/Down syndrome	292 (100)	244 (99.6)	241 (98.4)	270 (96.8)	.004
HIV/AIDS	278 (95.2)	237 (96.7)	234 (95.5)	271 (97.1)	.589

Most participants were male (79.2%-90.3%), had ≥10 years of experience, and worked in a hospital setting. Most were board certified in their main area of clinical practice, and over 99% of them were of Japanese nationality. There were no substantial differences when comparing the characteristics of the total population with those of participants who were aware of cCMVi (data not shown).

### 3.2 Survey outcomes

#### 3.2.1 Awareness

Awareness of cCMVi was highest among pediatricians (97.6%) and obstetricians (97.1%), followed by otolaryngologists (89.4%; Table 2). Pediatricians and obstetricians had the highest reported awareness for all other congenital conditions assessed except human immunodeficiency virus/acquired immunodeficiency syndrome. Otolaryngologists were relatively less aware of parvovirus B19 infection, group B streptococcal infection, congenital toxoplasmosis, and fetal alcohol syndrome. Internists were the least aware of other childhood infections, with the exception of congenital rubella syndrome, trisomy 21/Down syndrome, and human immunodeficiency virus/acquired immunodeficiency syndrome.

#### 3.2.2 Knowledge

Pediatricians and obstetricians were generally more knowledgeable about cCMVi compared with otolaryngologists and internists (Table 3). Overall, kissing and blood contact were the most frequently correctly identified potential CMV transmission routes (40.6%-65.6%). Pediatricians answered correctly more often than the other physician groups. However, correct identification of potential transmission routes was not very high, even among pediatricians, and ranged from 36.8% for changing diapers to 65.6% for kissing. Most physicians were aware that immunocompetent adults either had no symptoms or had elevated liver enzymes. Pediatricians and otolaryngologists were the most aware of hearing loss as a symptom of cCMVi in newborns (approximately 80%); awareness of this symptom was lower among obstetricians and internists. Of note, a large percentage of participants across all physician groups incorrectly identified congenital heart disease as a symptom in newborns (31.9%-43.4%), and cardiac problems as long-term effects of infection (20.2%-36.8%). Pediatricians and obstetricians were more aware of which behaviors can reduce the risk of CMV infection than otolaryngologists and internists.

**Table 3:** Knowledge of congenital cytomegalovirus infection among physicians who were aware of congenital cytomegalovirus infection.

	Pediatricians n = 285	Obstetricians n = 238	Otolaryngologists n = 219	Internists n = 193	*P* value^∗^
	n (%)				
Potential transmission route (horizontal)					
True					
Kissing	187 (65.6)	154 (64.7)	89 (40.6)	97 (50.3)	<.001
Changing diapers	105 (36.8)	116 (48.7)	36 (16.4)	30 (15.5)	<.001
Breast milk	158 (55.4)	79 (33.2)	78 (35.6)	102 (52.8)	<.001
Blood contact	184 (64.6)	121 (50.8)	121 (55.3)	116 (60.1)	.011
Sexual intercourse	163 (57.2)	106 (44.5)	100 (45.7)	108 (56.0)	.005
False					
Air conduction	18 (6.3)	18 (7.6)	9 (4.1)	8 (4.1)	.317
Direct skin contact	78 (27.4)	87 (36.6)	23 (10.5)	36 (18.7)	<.001
Do not know	11 (3.9)	17 (7.1)	35 (16.0)	22 (11.4)	<.001
Symptoms in immunocompetent adults					
True					
No symptoms	226 (79.3)	175 (73.5)	119 (54.3)	118 (61.1)	<.001
Elevated liver enzymes	175 (61.4)	114 (47.9)	78 (35.6)	104 (53.9)	<.001
False					
Cardiac problems	30 (10.5)	33 (13.9)	45 (20.5)	39 (20.2)	.004
Visual problems	40 (14.0)	40 (16.8)	49 (22.4)	55 (28.5)	<.001
Do not know	1 (0.4)	6 (2.5)	22 (10.0)	9 (4.7)	<.001
Symptoms in newborns					
True					
No symptoms	144 (50.5)	92 (38.7)	45 (20.5)	57 (29.5)	<.001
Jaundice	184 (64.6)	91 (38.2)	58 (26.5)	64 (33.2)	<.001
Elevated liver enzymes	238 (83.5)	131 (55.0)	77 (35.2)	96 (49.7)	<.001
Microencephaly	230 (80.7)	171 (71.8)	106 (48.4)	92 (47.7)	<.001
Intrauterine growth retardation	242 (84.9)	177 (74.4)	72 (32.9)	86 (44.6)	<.001
Hearing loss	228 (80.0)	154 (64.7)	173 (79.0)	81 (42.0)	<.001
Seizures	183 (64.2)	75 (31.5)	52 (23.7)	52 (26.9)	<.001
False					
Congenital heart disease	120 (42.1)	76 (31.9)	95 (43.4)	64 (33.2)	.016
Macrosomia	13 (4.6)	6 (2.5)	19 (8.7)	21 (10.9)	<.001
Renal problem	54 (18.9)	25 (10.5)	24 (11.0)	23 (11.9)	.018
Anal atresia	12 (4.2)	6 (2.5)	13 (5.9)	21 (10.9)	.002
Club foot	23 (8.1)	6 (2.5)	11 (5.0)	12 (6.2)	.040
Do not know	4 (1.4)	7 (2.9)	18 (8.2)	40 (20.7)	<.001
Long-term effects of cCMVi					
True					
Hearing loss	259 (90.9)	195 (81.9)	190 (86.8)	127 (65.8)	<.001
Developmental delay	256 (89.8)	175 (73.5)	139 (63.5)	113 (58.5)	<.001
Motor delay	218 (76.5)	132 (55.5)	91 (41.6)	79 (40.9)	<.001
Epilepsy	211 (74.0)	86 (36.1)	65 (29.7)	67 (34.7)	<.001
Visual problems	137 (48.1)	93 (39.1)	79 (36.1)	75 (38.9)	.033
False					
Cardiac problems	100 (35.1)	48 (20.2)	67 (30.6)	71 (36.8)	<.001
Obesity	16 (5.6)	7 (2.9)	9 (4.1)	16 (8.3)	.081
Increased risk for malignancy	18 (6.3)	8 (3.4)	15 (6.8)	19 (9.8)	.052
Diabetes	15 (5.3)	11 (4.6)	12 (5.5)	23 (11.9)	.016
Do not know	4 (1.4)	20 (8.4)	16 (7.3)	33 (17.1)	<.001
Behaviors to reduce the risk of CMV infection					
True					
Wash hands after diaper changing^†^	154 (54.0)	160 (67.2)	78 (35.6)	85 (44.0)	<.001
Avoid kissing young children on the mouth^†^	211 (74.0)	179 (75.2)	98 (44.7)	103 (53.4)	<.001
Do not share food, drink, or cutlery with young children^†^	200 (70.2)	168 (70.6)	97 (44.3)	93 (48.2)	<.001
Use a condom during sexual intercourse	136 (47.7)	80 (33.6)	94 (42.9)	91 (47.2)	.005
False					
Cook meat thoroughly	41 (14.4)	61 (25.6)	31 (14.2)	33 (17.1)	.004
Avoid cats	52 (18.2)	46 (19.3)	35 (16.0)	34 (17.6)	.827
Avoid unpasteurized dairy products	42 (14.7)	51 (21.4)	31 (14.2)	32 (16.6)	.141
Do not know	28 (9.8)	28 (11.8)	57 (26.0)	46 (23.8)	<.001

#### 3.2.3 Actual and preferred clinical practices on education, screening, and follow-up

**Figure 1:**
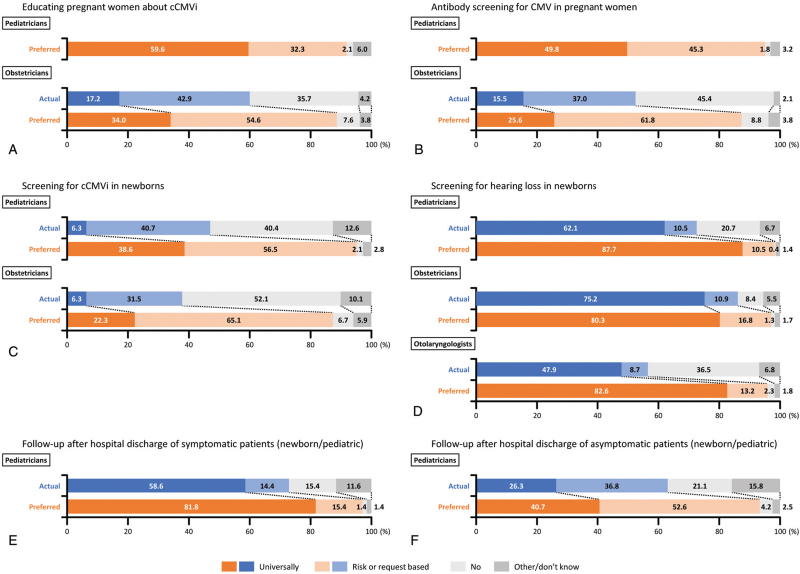
Actual and preferred clinical practices related to cCMVi education, screening, and follow-up among physicians who were aware of cCMVi. (A) Educating pregnant women about cCMVi (pediatricians and obstetricians). (B) Antibody screening for CMV in pregnant women (pediatricians and obstetricians). (C) Screening for cCMVi in newborns (pediatricians and obstetricians). (D) Screening for hearing loss in newborns (pediatricians, obstetricians, and otolaryngologists). (E) Follow-up after hospital discharge of symptomatic patients (newborn/pediatric; pediatricians only). (F) Follow-up after hospital discharge of asymptomatic patients (newborn/pediatric; pediatricians only). cCMVi = congenital cytomegalovirus infection, CMV = cytomegalovirus.

Figure 1 shows physicians’ actual and preferred clinical practices regarding cCMVi education, screening, and follow-up. Approximately 90% of pediatricians and obstetricians answered that their preferred practice would include educating either all (universal) or some (based on risk or request) pregnant women about cCMVi; however, only 60.1% of obstetricians answered that they actually were able to do so in their current practice (Fig. 1A). A similar discrepancy was observed for antibody screening for CMV in pregnant women. Approximately 90% of pediatricians and obstetricians preferred to screen, but only 52.5% of obstetricians did so in their actual practice (Fig. 1B). Although rarely performed in actual practice, nearly 40% of pediatricians considered universal screening of newborns for cCMVi to be necessary. Screening for cCMVi in newborns (universally + based on risk or request) was thought to be necessary by 95.1% of pediatricians and 87.4% of obstetricians (Fig. 1C). Although a high proportion of the study population performed universal screening for hearing loss in newborns in their actual practice, physicians answered that it would be preferable to screen more frequently (Fig. 1D). In actual practice, follow-up of symptomatic patients (newborn/pediatric) after hospital discharge was reported by 73.0% of pediatricians (universally + based on risk or request); however, as preferred practice, nearly all respondents (97.2%) answered that they would follow up (Fig. 1E). Findings regarding follow-up of asymptomatic patients (newborn/pediatric) after hospital discharge were similar. Approximately 60% of pediatricians reported that they followed up asymptomatic patients in their actual clinical practice (universall + based on risk or request), but over 90% considered that follow-up (similar to the follow-up received by symptomatic patients) was preferable (Fig. 1F).

#### 3.2.4 Feasibility of preventative behaviors

**Figure 2:**
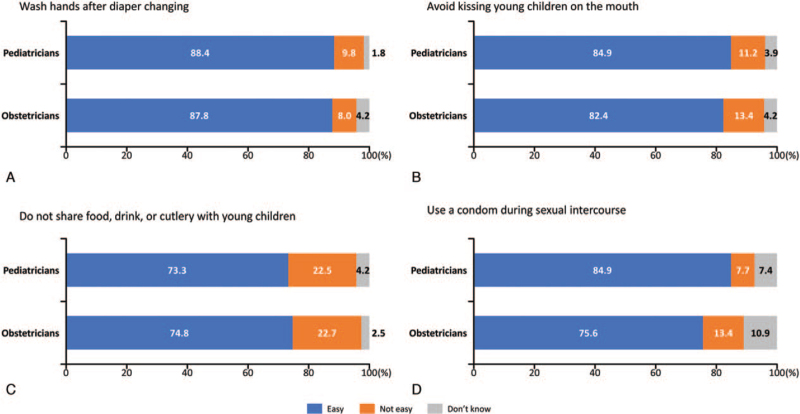
Pediatrician and obstetrician (aware of cCMVi) perceptions regarding the feasibility for pregnant women to practice preventative behaviors. (A) Wash hands after diaper changing. (B) Avoid kissing young children on the mouth. (C) Do not share food, drink, or cutlery with young children. (D) Use a condom during sexual intercourse. cCMVi = congenital cytomegalovirus infection.

Most physicians (73.3%-88.4% of pediatricians or obstetricians) considered preventative behaviors to be easy to perform during pregnancy (Fig. 2).

#### 3.2.5 Other findings

Few physicians were aware of the leaflets on mother-to-child infection distributed by the TOACH-no-Kai patient group and a research group funded by the Agency for Medical Research and Development. Only 22.1% and 21.8% of pediatricians, 12.2% and 11.3% of obstetricians, 10.4% and 10.9% of internists, and 8.2% and 5.5% of otolaryngologists were aware of the leaflets from the patient group and the research group, respectively.

Among obstetricians, 3.8% reported that they had experienced a patient who decided to receive an abortion due to a CMV infection (including suspected case) during pregnancy.

## 4 Discussion

Overall, there was a high level of awareness of cCMVi among physicians in Japan. However, importantly, we found that knowledge regarding cCMVi among physicians could be improved. To our knowledge, our study is the first to report a discrepancy between actual and preferred clinical practices with regard to cCMVi. Further, we found that most physicians consider that preventative behaviors can be easily implemented.

In contrast to the high incidence of cCMVi globally,^[2,12–15]^ awareness on cCMVi is quite limited among pregnant women and the general public.^[20,23]^ To effectively educate pregnant women about the disease and preventative behaviors, and to perform appropriate screening and follow-up, it is important for physicians to have an adequate level of knowledge regarding cCMVi. The amount and quality of data specifically describing the level of detailed knowledge possessed by physicians in Japan can be improved.

We found that recognition of cCMVi was high among physicians; however, knowledge about the disease can be improved. In addition to the limited proportion of physicians who could correctly answer the survey questions, some incorrect answers were considered as correct by a number of physicians, including pediatricians and obstetricians. Similarly, surveys of healthcare professionals outside of Japan have indicated insufficient detailed knowledge of cCMVi symptoms in newborns^[25,26]^ and CMV transmission routes.^[25,26,28,30]^

Previous studies (outside of Japan) have reported that most healthcare professionals do not counsel expectant mothers on the risks and preventative behaviors for cCMVi.^[28–30]^ The present survey not only assessed actual practice regarding education of pregnant women, but also physicians’ preferred practice. Most importantly, the survey results indicated a discrepancy between actual and preferred clinical practices that had not previously been investigated. Physicians (mainly pediatricians and obstetricians) thought that there was a need to educate pregnant women about cCMVi and to increase screening and follow-up. This discrepancy between actual and preferred practices may be attributable to multiple factors, including the weak financial incentives in current insurance reimbursement schemes for clinics and hospitals to perform education and antibody screening in pregnant women and/or the chronic shortage of professional human resources within obstetrics and pediatric departments in Japan.^[34]^

This survey revealed that physicians believe it would be easy for pregnant women to practice behaviors that can reduce the risk of CMV transmission. Although the perceptions of pregnant women should also be evaluated, this information can help guide the development of educational materials on preventative behaviors for cCMVi.

## 5 Limitations

Our study has several limitations. First, the generalizability of the findings may be limited. For example, the proportion of female physicians who responded to our survey (between 9.7% and 20.8%) was somewhat lower than expected, as approximately 22% of physicians in Japan are documented to be women.^[35]^ Additionally, most respondents had ≥10 years of experience as a physician. These proportions within our survey population may be attributable to several factors, including the method used to assemble the target panel and differences in the tendencies of physicians of different sexes or age groups to answer online surveys. Such factors may have biased our findings. Second, the newly developed questionnaire used in the survey, although based on prior published studies, was not validated. The data should be interpreted with caution; the wording may have been ambiguous in some survey items, including the knowledge questions (e.g., changing diapers itself does not necessarily mean a direct risk of infection while contact with infected urine does) and the definition of “preferred” clinical practice. Third, the results of the self-administered web-based survey were subject to non-response bias, which may have resulted in overestimation of awareness or knowledge levels (of the 4890 potential participants recruited, 3617 did not access the survey). Survey respondents, especially completers, may have been more likely to be interested in health or the study contents. Finally, newborn screening for high-risk infants became available in Japan starting in 2018 and its cost is reimbursable^[36]^; this may have influenced our results.

## 6 Conclusions

In conclusion, this was the first survey of physicians in Japan regarding cCMVi awareness, knowledge, actual and preferred clinical practices, and experience. This study indicates that there is a high awareness of cCMVi, but that knowledge on the transmission route, symptoms, long-term effects, and preventative behaviors of cCMVi among Japanese physicians can be improved. It also revealed a discrepancy between actual and preferred clinical practices. Our findings herein will inform experts and researchers and potentially help to guide them toward the development of effective strategies for cCMVi prevention and screening.

## Acknowledgments

The authors wish to thank Yukari Ohta and Masao Fukushima of MSD K.K., Tokyo, Japan, and Anushua Sinha, Louis R. Macareo, Wei Wang, Elizabeth Goodman, and Lindsay Hermany of Merck & Co., Inc., Kenilworth, NJ, USA, for their substantial contributions to the study. The web-based survey was operated by Masakazu Nishikori and Toshiaki Murofushi of INTAGE Healthcare Inc., Tokyo, Japan, and statistical support was provided by Takayuki Sawada of Clinical Study Support, Inc., Nagoya, Japan. The authors also wish to thank Sarah Bubeck, PhD, of Edanz (www.edanz.com) for providing medical writing support, which was funded by MSD K.K., Tokyo, Japan, in accordance with Good Publication Practice (GPP3) guidelines (http://www.ismpp.org/gpp3).

## Author contributions

**Conceptualization:** Aya Okahashi, Masayuki Kobayashi, Naomi Hiraishi, Ichiro Morioka.

**Formal analysis:** Masayuki Kobayashi, Kotoba Okuyama.

**Data interpretation:** Aya Okahashi, Masayuki Kobayashi, Ichiro Morioka.

**Methodology:** Aya Okahashi, Masayuki Kobayashi, Naomi Hiraishi, Ichiro Morioka.

**Writing – original draft:** Aya Okahashi, Masayuki Kobayashi, Ichiro Morioka.

**Writing – review & editing:** Aya Okahashi, Masayuki Kobayashi, Naomi Hiraishi, Ichiro Morioka, Kotoba Okuyama.

## Correction

Figure 1 has been replaced with a higher resolution version. Nothing else about the figure has been changed.

## Supplementary Material

Supplemental Digital Content
