# Supplementary figures and images for: Survey of physician knowledge of congenital cytomegalovirus infection and clinical practices in Japan: A web-based survey
Source: Medicine (Baltimore). 2021 Nov 5;100(44):e27589. doi: 10.1097/MD.0000000000027589 (PMC8568346; doi:10.1097/MD.0000000000027589)

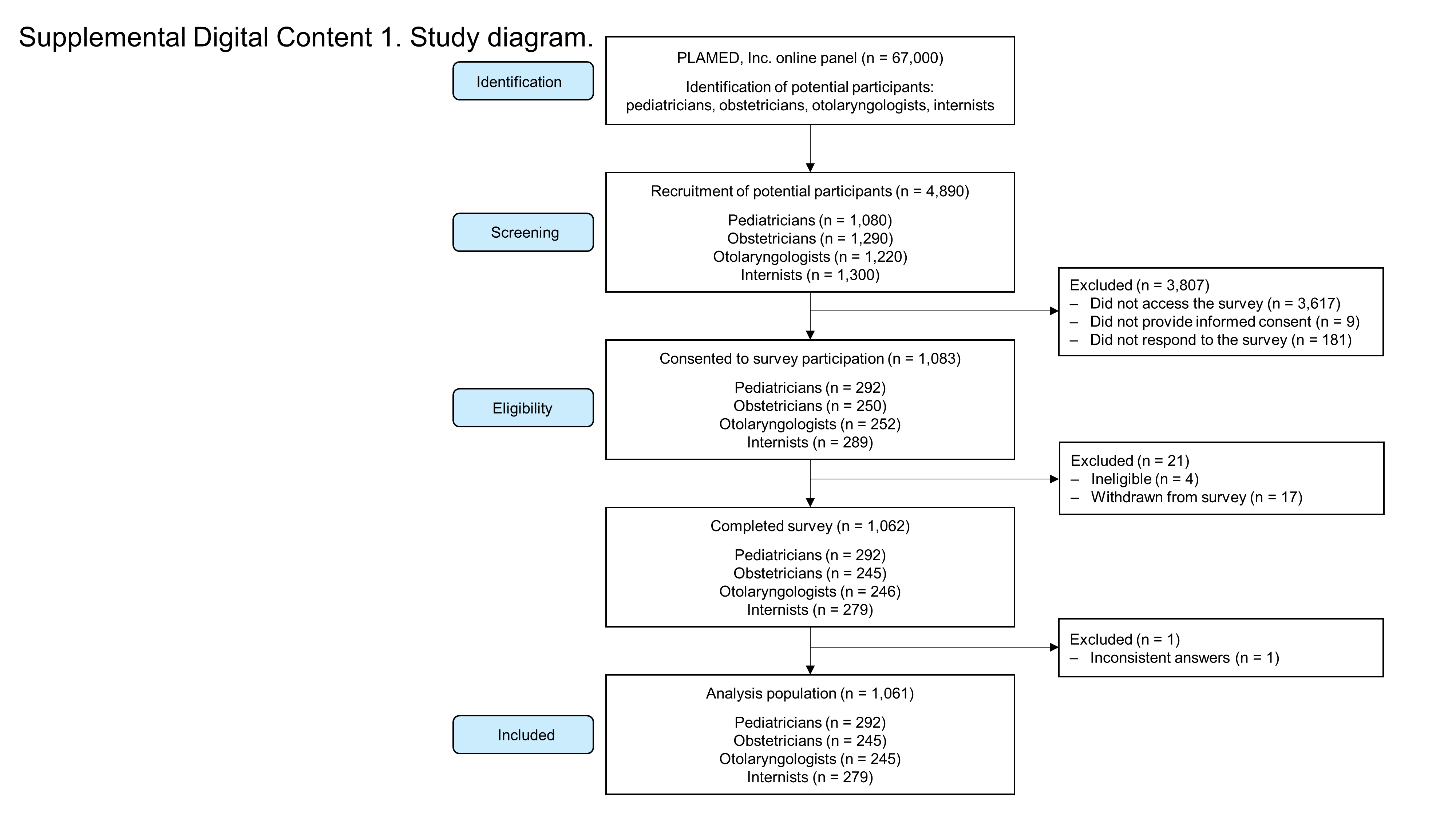


**Supplemental Digital Content 1.** Study diagram.

Supplement: Supplemental Digital Content [file medi-100-e27589-s001.doc]
